# MiR-185-5p Protects Against Angiogenesis in Polycystic Ovary Syndrome by Targeting VEGFA

**DOI:** 10.3389/fphar.2020.01030

**Published:** 2020-07-15

**Authors:** Jingzan Wei, Yanyan Zhao

**Affiliations:** ^1^ Department of Management, Shengjing Hospital of China Medical University, Shenyang, China; ^2^ Department of Clinical Genetics, Shengjing Hospital of China Medical University, Shenyang, China

**Keywords:** Polycystic Ovary Syndrome, miR-185-5p, vascular endothelial growth factor A, angiogenesis, human ovarian microvascular endothelial cells

## Abstract

Polycystic Ovary Syndrome (PCOS) is a heterogeneous endocrine disease with high incidences in women of reproductive age. Although miR-185-5p (miR-185) was decreased in PCOS patients, the exact function of miR-185 on PCOS development still requires further investigation. In this study, rat injected with dehydroepiandrosterone (DHEA) was established as a PCOS model. A lentivirus carrying miR-185 was employed to examine its effect on PCOS symptoms. Then we performed the luciferase reporter assay to validate the interactions between miR-185 and vascular endothelial growth factor A (VEGFA). Finally, human ovarian microvascular endothelial cells (HOMECs) were induced by VEGF to explore the role of miR-185 in the angiogenic process. The results showed that miR-185 overexpression improved insulin level alteration and ovarian histological lesion in PCOS rats. We also found that miR-185 reduced the excessive angiogenesis as indicated by alterations of VEGFA, ANGPT1/2, PDGFB/D, α-SMA and CD31 in the ovary of PCOS rats. Luciferase reporter assay identified that VEGFA directly interacted with miR-185, and its expression level was negatively regulated by miR-185. The *in vitro* results further demonstrated that miR-185-induced suppression of cell proliferation, migration and tube formation was attenuated by VEGF in HOMECs. In summary, this is the first study to show that miR-185 can target VEGFA to inhibit angiogenesis, thus improving the development of PCOS. These findings develop a molecular candidate for PCOS prevention and therapy.

## Introduction

Polycystic Ovary Syndrome (PCOS), a prevalent endocrine disorder, has nearly 6–10% incident rates in women of reproductive age. The characteristics of PCOS predominantly reflect in menstrual disturbance, polycystic ovaries and high androgen levels, which may cause infertility and estrogen-dependent tumors. In addition, PCOS also increases the occurrences of metabolic disorders, including hyperandrogenism, insulin resistance, type 2 diabetes mellitus and cardiovascular disease (Stepto et al., 2013). However, the pathogenesis of PCOS remains confusing due to its heterogeneous nature.

PCOS patients who undergo over response to gonadotrophin stimulation can trigger severe ovarian hyperstimulation syndrome (OHSS) (Peitsidis and Agrawal, 2010). Evidence suggests the involvement of abnormal ovarian angiogenesis in various pathological conditions of PCOS, such as OHSS, ovulation disorder, subfertility and even endometriosis (Geva and Jaffe, 2000; Reynolds et al., 2002; Di Pietro et al., 2018). Notably, a current study from Xie et al. shows an evident correlation between PCOS progress and the dysregulation of angiogenic factors (Xie et al., 2017). Much of literature in this field has highlighted that vascular endothelial growth factor (VEGF) may be a crucial mediator of OHSS (McClure et al., 1994; Ferrara and Davis-Smyth, 1997; Agrawal et al., 1998; Garcia-Velasco and Pellicer, 2003), which is high-expressed in PCOS patients (Peitsidis and Agrawal, 2010; Almawi et al., 2016). Di Pietro et al. have demonstrated that VEGF levels may play a part in the mediation of metformin on improving follicular development and ovarian over-angiogenesis in PCOS rats (Di Pietro et al., 2015). It also considers that targeting angiogenesis may be a promising therapeutic option for treating PCOS (Duncan and Nio-Kobayashi, 2013). Therefore, this paper is to further explore a potential mechanism about the involvement of angiogenesis in PCOS progress.

MicroRNA (miRNA) is known as a class of endogenous non-coding RNA with 22–24 nucleotides approximately that impacts complex pathophysiological processes (Ambros, 2001). As far as we know, miR-185-5p (miR-185) is firstly identified to control cell growth in human lung cancers (Takahashi et al., 2009). A growing body of studies suggests that miR-185 may also contribute to regulate a series of diseases, such as lipid metabolism disorder, neurological disorder, liver fibrosis, dilated cardiomyopathy and idiopathic pulmonary fibrosis (Forstner et al., 2014; Wang et al., 2014; Lei et al., 2016; Li et al., 2016; Yu et al., 2016). Evidence from Xu et al. shows apparent decrease of miR-185 expression in the cumulus granulosa cells of PCOS patients (Xu et al., 2015). Furthermore, the reduction of miR-185 level was confirmed in the ovaries of PCOS rats in our preliminary experiments. Thus, these evidences indicate the potential involvement of miR-185 in PCOS progress. However, the exact function of miR-185 in regulating the pathogenesis of PCOS is yet to be elucidated. Interestingly, we screened that VEGFA might be a probable target gene of miR-185 through bioinformatics analysis. Therefore, this study was designed to explore whether miR-185 could target VEGFA to regulate PCOS pathogenesis.

In the current study, the classical PCOS model was locally injected with lentivirus carrying miR-185 to investigate the role of miR-185. Then VEGF-induced human ovarian microvascular endothelial cells (HOMECs) were established to further explore the underlying mechanism of miR-185 on angiogenesis.

## Materials and Methods

### Animal Treatment

In this work, ethical approval was approved by Shengjing Hospital of China Medical University. Animal procedures were performed based on the Guide for the Care and Use of Laboratory Animals. Female Sprague–Dawley (SD) rats (3-week old) were acquired from Liaoning Changsheng Biotechnology Company (Benxi, China) and had free access to get food and water. The rats were housed in 25 ± 1°C with a 12-h light/dark cycle under 45–55% humidity. The PCOS rat model was established as previous reported (Wang et al., 2017). In brief, the dehydroepiandrosterone (DHEA, D106380, Aladdin, Shanghai, China) with a concentration of 60 mg/kg was subcutaneously injected into rats daily for consecutive 3 weeks. Meanwhile, the Control rats (n = 12) were just administrated with an equal volume of sesame oil in the same manner. Then we measured the fasting blood glucose and fasting insulin levels to calculate the insulin resistance index at 12 h post fasting. The homeostasis model assessment of insulin resistance (HOMA-IR) was calculated as the formula: fasting insulin concentration (mlU/L) × fasting glucose concentration (mmol/L)/22.5. The PCOS rats (n = 36) with HOMA-IR >2.8 were selected for further experiments (Wang et al., 2017).

The PCOS rats were further randomly divided into three groups (n = 12 in each group): PCOS group, PCOS + NC group (PCOS rats infected with negative control lentivirus) and PCOS + rno-miR-185 group (PCOS rats infected with rno-miR-185 overexpressing lentivirus). The lentivirus overexpressing miR-185 or NC was constructed using the pScio lentiviral vector system (#11578) from Addgene (Watertown, MA, USA) that carried with an EGFP maker. In this process, rats were anesthetized with 50 mg/kg pentobarbital sodium and then shaved on the back skin. Both ovaries were isolated after cutting into a small incision on the back skin. Then both ovaries of PCOS rats were prepared to receive a subcaspsular injection of lentivirus overexpressing rno-miR-185 or NC at the concentration of 5 × 10^8^ TU/ml. Each side of the ovary was injected twice. After 2 weeks, the rats fasted 12 h were used to assess HOMA-IR and test serum insulin release. Following the above measurements, all animals at diestrus were sacrificed and the ovaries were harvested immediately for the following examinations. Six rats were used for histological analysis, and another six rats were prepared for the qRT-PCR, Western blot and ELISA examinations. Each experiment in animals was performed for 6 times.

### Insulin Release Assessment

For the detection of serum insulin release, the fasting rats were given glucose by gavage at a dose of 3 g/kg. The blood samples were collected from the orbital venous rapidly at 0, 30, 60 and 120 min after gavage.

### Cell Culture

Human ovarian microvascular endothelial cells (HOMECs) were obtained from Zhongqiaoxinzhou (Shanghai, China), and cultured in an endothelial cell medium (1001, Zhongqiaoxinzhou) containing with 5% fetal bovine serum (FBS; SH30084.03, Hyclone, South Logan, UT, USA) and 1% endothelial cell growth supplement (EGGS; 1052, Zhongqiaoxinzhou) in a 5% CO_2_ incubator at 37°C. HOMECs were infected with NC or hsa-miR-185-5p overexpressing lentivirus at a multiplicity of infection (MOI) of 20. Following the 72-h infection, cells were cultured with recombinant VEGF (100 ng/ml; APA143Hu01, USCN, Wuhan, China) (Palanisamy et al., 2019). All experiments *in vitro* were repeated for three times.

### Hematoxylin and Eosin (H&E) Staining

To evaluate the changes of ovarian morphology, the extracted ovaries were embedded in paraffin and sectioned into 5-μm slides. Then sections were applied to test with an H&E staining kit (WLA051a, Wanleibio, Shenyang, China) as the manufacturers described. All histological changes were observed by an optical microscope (BX53, OLUMPUS, Tokyo, Japan) and imaged by a camera device (DP73, OLUMPUS) at the magnification of ×40 or ×100.

### Quantitative Real-Time PCR (qRT-PCR)

In this process, we firstly isolated total RNAs by RNAsimple Total RNA kit (DP419, TIANGEN, Beijing, China) from the whole ovaries of rats or HOMECs. Following measuring the concentrations of extracted RNAs using a NANO 2000 ultraviolet spectrophotometer (Thermo Fisher Scientific, Waltham, MA, USA), the RNA samples were conducted to be reverse-transcribed into cDNAs by the use of M-MLV reverse transcriptase (NG212, TIANGEN). All designed primers in **Table 1** were synthesized by Sangon Biotech (Shanghai, China). The samples treated with SYBR Green (SY1020, Solarbio, Beijing, China) were employed to detect gene expression with a real-time PCR instrument (Exicycler96, BIONEER, Daejeon, Korea). Finally, the relative expression of VEGFA or miR-185 in rats or HOMECs was calculated using 2^−ΔΔCT^ method. VEGFA was normalized to GAPDH and miR-185 was normalized to 5S.

**Table 1:** Primer sequences used in this study.

Gene	Primer sequences	
rno/hsa-miR-185-5p	RT	GTTGGCTCTGGTGCAGGGTCCGAGGTATTCGCACCAGAGCCAACTCAGGA
	Forward	TGGAGAGAAAGGCAGTTCCTGA
	Reverse	TGCAGGGTCCGAGGTATT
5S	RT	GTTGGCTCTGGTGCAGGGTCCGAGGTATTCGCACCAGAGCCAACAAAGCCTAC
	Forward	GATCTCGGAAGCTAAGCAGG
	Reverse	TGCAGGGTCCGAGGTATTCG
rat VEGFA	Forward	ATCCTGGAGCGTTCACTG
	Reverse	TCACCGCCTTGGCTTGTC
rat GAPDH	Forward	ACGTTGACATCCGTAAAGAC
	Reverse	TAGGAGCCAGGGCAGTAA
homo VEGFA	Forward	GAAGGAGGAGGGCAGAAT
	Reverse	CACAGGATGGCTTGAAGAT
homo GAPDH	Forward	GACCTGACCTGCCGTCTAG
	Reverse	AGGAGTGGGTGTCGCTGT

### Enzyme-Linked Immunosorbent Assay (ELISA)

The blood insulin level was detected by an insulin ELISA kit (CEA448Ra, USCN). In addition, the extracted whole ovaries were homogenized, and the supernatants were quantified using a BCA assay kit (PC0020, Solarbio). Then the ovarian proteins were collected to test the concentrations of key angiogenic factors using commercial ELISA kits, including VEGFA (EK3832/2, Multi Sciences, Hangzhou, China), ANGPT1 (SEA008Ra, USCN), ANGPT2 (SEA009Ra, USCN), PDGFB (SEC921Ra, USCN) and PDGFD (SEC919Ra, USCN). The optical density at 450 nm was recorded using a microplate (ELX-800, BioTek, Biotek Winooski, Vermont, USA).

### Immunohistochemistry

The ovarian tissue slices as above mentioned was utilized to immunohistochemistry examination. Sections were incubated with primary antibodies against α-SMA (55135-1-AP, Proteintech, Wuhan, China) or CD31 (A11525, Abclonal, Wuhan, China) overnight at 4°C. After washing in PBS, slides stained with α-SMA were incubated with goat against rabbit biotin-labeled antibody (A0277, Beyotime, Shanghai, China) in PBS solution for 60 min at 37°C and conjugated with HRP-labeled Streptavidin (A0303, Beyotime) for 30 min at room temperature. The HRP-labeled goat anti-rabbit antibody (#31460, Thermo Fisher Scientific) was prepared to conjugate with CD31. Then slides were colorated using DAB reagent and counterstained with hematoxylin. The images were shot at × 100 or ×400 magnification.

### Western Blot

Protein samples from HOMECs were extracted and quantified for determining protein level. Samples were subjected to SDS-PAGE analysis and electrotransferred into PVDF membrane (IPVH00010, Millipore, Billerica, MA, USA). Membranes were conducted to incubate with primary antibodies against VEGFA (A17000, Abclonal) and GAPDH (60004-1-Ig, Proteintech) overnight at 4°C. Then HRP-conjugated goat anti-rabbit antibody (SE134, Solarbio) or goat anti-mouse antibody (SE131, Solarbio) was used to incubate with membranes. Finally, the blots were developed with ECL substrate reagent (PE0010, Solarbio). The optical density of target proteins was quantified with Gel-Pro-Analyzer software (Media Cybernetics, Silver Springs, MD, USA). GAPDH was considered as an internal control of VEGFA.

### MTT

Following the lentiviral infection and drug treatment, HOMECs were applied to undergo MTT assay (KGA311, KeyGen, Nanjing, China) according to manufacturer’s instruction at 0, 24, 48, 72 and 96 h of culture, respectively. In brief, cells were treated with MTT for 5 h at 37°C, and then the absorbance was read with a microplate reader at 570 nm.

### Transwell Assay

Transwell assay was performed using a 24-well transwell inserts (3422, Corning Incorporated, Corning, NY, USA). The collected HOMECs were resuspended in a serum-free medium and plated into the upper chamber at the density of 1× 10^4^ cells per well. The lower chambers were added with 30% FBS. After incubation for 24 h or 48 h, HOMECs were fixed in 4% paraformaldehyde and incubated with crystal violet (0528, Amresco, Solon, OH, USA). The observation of migratory cells was conducted using a phase-contrast microscopy (IX53, OLUMPUS) under ×200 magnification. Finally, the number of cells migrated into lower chambers was counted from five fields randomly per well.

### Tube Formation Assay

For testing the tube formation, Matrigel (MA01730, BD Biosciences, San Jose, CA, USA) was applied in this step. Cells were seed at the 96-well matrigel and maintained for 24 h at 37°C. Then the phase-contrast microscopy was used to observe tube formation at ×100 magnification.

### Luciferase Activity Assay

Luciferase reporter assay was conducted to validate the binding activity between miR-185 and VEGFA. The pmirGLO vector (E133A, Promega, Madison, WI, USA) was used to construct wild type (wt) or point mutant type (mut) VEGFA luciferase reporter vector from human and rat. The cotransfection of VEGFA vector (wt or mut) and miR-185 mimics or NC mimics (GenePharma, Shanghai, China) in 293T cells were mediated by Lipofectamine 2000 (11668-019, Invitrogen, Carlsbad, CA, USA) for 48 h. Finally, the commercial dual luciferase reporter assay kit (KGAF040, KeyGen) was used to measure the binding activity of miR-185 by fly luciferase activity/renilla luciferase activity.

### Statistical Analysis

All results were shown as mean ± SD, and the management and analysis of data were carried out with GraphPad Prism software. Differences in two groups were analyzed using unpaired t test. One-way ANOVA or Repeated measures ANOVA following Bonferroni’s *post hoc* test was utilized to assess the statistical significance among multiple groups. P values less than 0.05 was thought as significantly statistical difference.

## Results

### MiR-185 Ameliorated the Insulin Release in PCOS Rats

**Figure 1:**
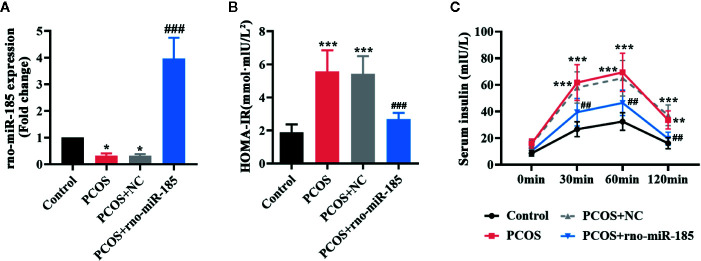
MiR-185 ameliorated the insulin release in PCOS model. **(A)** Relative levels of miR-185 were decreased in the ovaries of PCOS rats and increased by its specific lentivirus. **(B, C)** The HOMA-IR value **(B)** and serum insulin level **(C)** in PCOS rats were suppressed by miR-185. *p < 0.05, **p < 0.01, ***p < 0.001 vs Control. ^##^p < 0.01, ^###^p < 0.001 vs PCOS + NC.

After 3 weeks of DHEA injections, the HOMA-IR index of rats was measured, and HOMA-IR >2.8 was selected as a PCOS rat model. The images of **Supplementary Figure 1** indicated that there was strong green fluorescence in the ovaries infected with NC or miR-185 lentivirus. However, no fluorescence was observed in the ovaries of Control or PCOS rats. Furthermore, as shown in **Figure 1A**, miR-185 level was significantly reduced in the ovary of PCOS rats, and as it is expected to be increased by the injection with ectopic lentivirus. The results suggested the successful infection of the lentivirus overexpressing miR-185 on the ovaries. To determine the effect of miR-185 in PCOS rats, the HOMA-IR was firstly measured. Overexpression of miR-185 could restore the high level of HOMA-IR in PCOS rats (**Figure 1B**). Then the serum insulin release at indicated time points following glucose treatment was tested, demonstrating that the remarkable upregulation of insulin release level in PCOS rats were downregulated by miR-185 (**Figure 1C**).

### MiR-185 Attenuated Ovarian Morphological Damages in PCOS Rats

**Figure 2:**
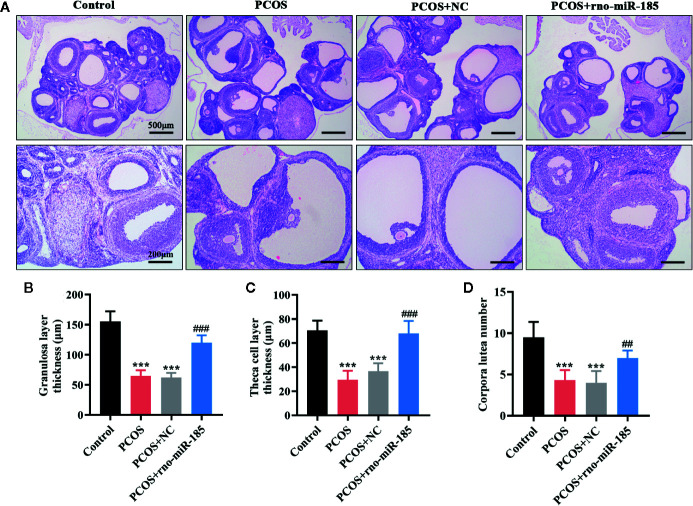
MiR-185 attenuated ovarian morphological damages in PCOS model. **(A)** Representative images of HE staining for the ovarian lesions in PCOS rats. Upper panel: Scale bar = 500 μm; Lower panel: Scale bar = 200 μm. **(B–D)** Quantitative results for the thickness of granulosa layer **(B)** and theca cell layer **(C)**, and the number of corpora lutea **(D)**. ***p < 0.001 vs Control. ^##^p < 0.01, ^###^p < 0.001 vs PCOS + NC.

To observe the ovarian histological changes, HE staining was conducted. It was apparent from **Figure 2A** that ovarian structural integrity, multiple corpora lutea, and ovarian follicles in different stages were presented in the Control rats. However, the larger cystic follicles, thinner granulosa and theca cell layers, and decreased corpora lutea as well as the disappearance of oocyte and corona radiate were observed in ovaries of PCOS rats. Overexpression of miR-185 attenuated the structural lesions in the ovaries of PCOS rats. Furthermore, the quantitative analyses from **Figures 2B–D** also demonstrated that miR-185 could protect against the decreases of granulosa cell layer thickness, theca cell layer thickness, and corpora lutea number in PCOS rats.

### MiR-185 Inhibited the Angiogenic Effect in the Ovary of PCOS Rats

**Figure 3:**
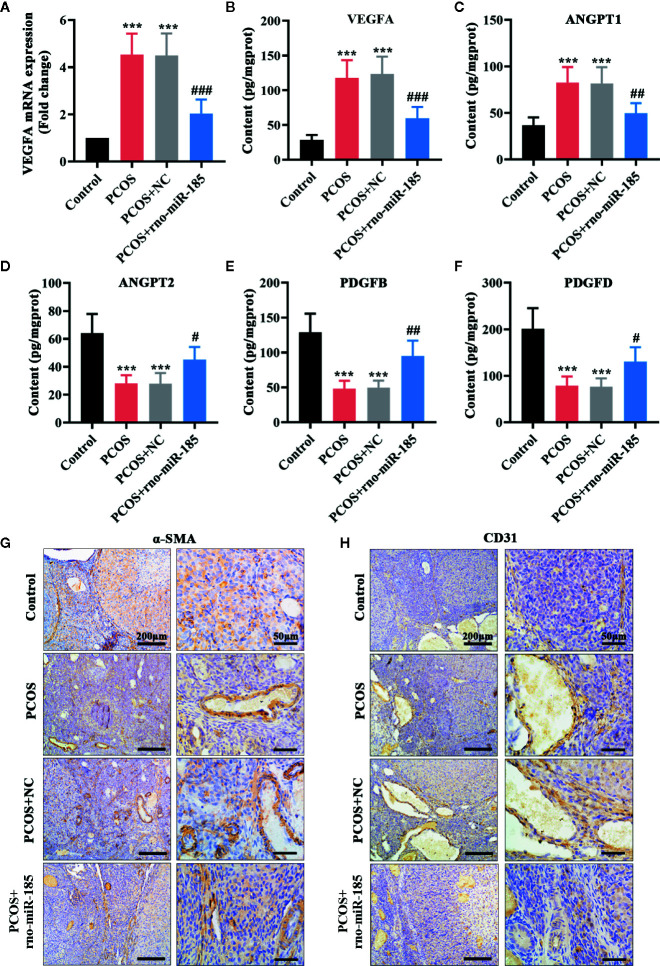
MiR-185 inhibited the angiogenic effect in the ovaries of PCOS rats. **(A)** Relative mRNA levels of VEGFA were tested using qRT-PCR. **(B–F)** The concentrations of VEGFA **(B)**, ANGPT1 **(C)**, ANGPT2 **(D)**, PDGFB **(E)** and PDGFD **(F)** were measured by ELISA. **(G, H)** The immunopositive staining of α-SMA **(G)** and CD31 **(H)** were examined with immunohistochemistry. Left panel: Scale bar = 200 μm; Right panel: Scale bar = 50 μm. ***p < 0.001 vs Control. ^#^p < 0.05, ^##^p < 0.01, ^###^p < 0.001 vs PCOS + NC.

The results in **Figure 3** were set out to assess ovarian angiogenesis in PCOS rats. As shown in **Figures 3A, B**, PCOS rats showed significant increase of VEGFA mRNA and protein, but overexpression of miR-185 attenuated the increase of VEGFA in PCOS model. Further, the alterations of angiogenesis-associated factors in the ovary were tested using ELISA (**Figures 3C–F**). Comparing with the control rats, PCOS rats induced remarkable increase of ANGPT1, and decrease of ANGPT2, PDGFB and PDGFD. However, miR-185 overexpression reversed the protein alterations of angiogenesis-associated factors. In addition, we observed that the increased immunopositive staining of α-SMA and CD31 in the ovarian vessel of PCOS rats was restored by overexpressing miR-185 (**Figures 3G, H**), indicating that miR-185 not only modulated periendothelial cells but also affected endothelial cells in ovaries. Taken together, the data implied that miR-185 had an anti-angiogenic effect on the ovary of PCOS rats.

### MiR-185 Directly Interacted With VEGFA

**Figure 4:**
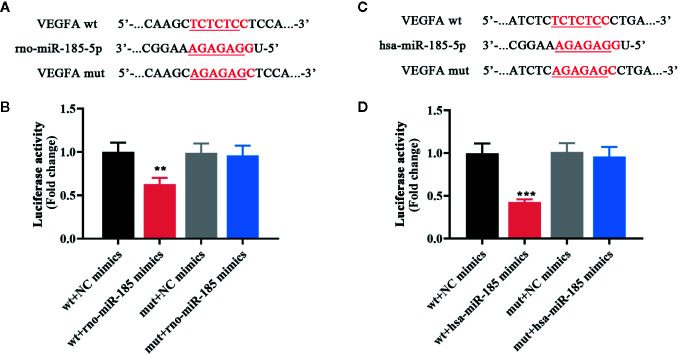
MiR-185 directly targeted VEGFA. **(A)** The putative binding sites of rno-miR-185-5p with VEGFA. **(B)** Relative luciferase activity between rno-miR-185-5p and VEGFA was detected. **(C)** The putative binding sites of hsa-miR-185-5p with VEGFA. **(D)** Relative luciferase activity between hsa-miR-185-5p and VEGFA was detected. Red sequences: binding sites. Underlined sequences: mutant sites. **p < 0.01, ***p < 0.001 vs wt + NC mimics.

Since the results in **Figure 3** demonstrated that miR-185 could block the ovarian angiogenesis of PCOS and negatively regulate VEGFA expression, we speculated that it might be complementary with the 3’-UTR of VEGFA. The putative complementary sequences between rno/has-miR-185 and VEGFA were shown in **Figures 4A, C**. Then luciferase reporter assay was performed to explore the associations between miR-185 with VEGFA. Data from **Figures 4B, D** indicated that there was a significant difference between VEGFA-wt + NC mimics and VEGFA-wt + miR-185 mimics. However, no significant change of luciferase activity was found between VEGFA-mut + NC mimics and VEGFA-mut + miR-185 mimics. The results showed that VEGFA was a potential target gene of miR-185.

### Overexpression of miR-185 Reduced VEGFA Expression in HOMECs

**Figure 5:**
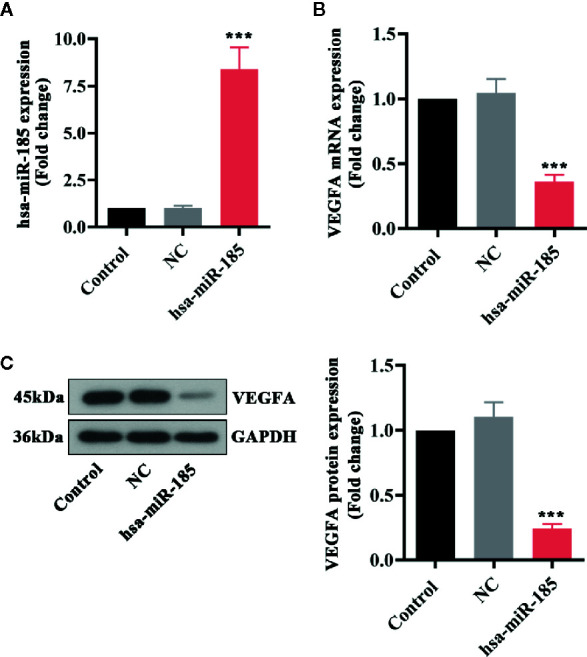
Overexpression of miR-185 reduced VEGFA expression in HOMECs. **(A)** Relative levels of miR-185 were elevated by its specific lentivirus in HOMECs. **(B, C)** Relative VEGFA mRNA **(B)** and protein **(C)** levels were decreased by upregulating miR-185 in HOMECs. ***p < 0.001 vs NC.

Furthermore, several *in vitro* experiments were designed to better uncover the underlying mechanism of miR-185 in PCOS. The results in **Figure 5A** showed that there was an obvious increase of miR-185 in HOMECs by its overexpressing lentivirus. Furthermore, the mRNA and protein expression levels of VEGFA were significantly down-regulated by miR-185 in HOMECs (**Figures 5B, C**). These data further confirmed the direct regulation of miR-185 on VEGFA expression.

### VEGF Mediated the Inhibition of miR-185 on Cell Proliferation, Migration and Tube formation in HOMECs

**Figure 6:**
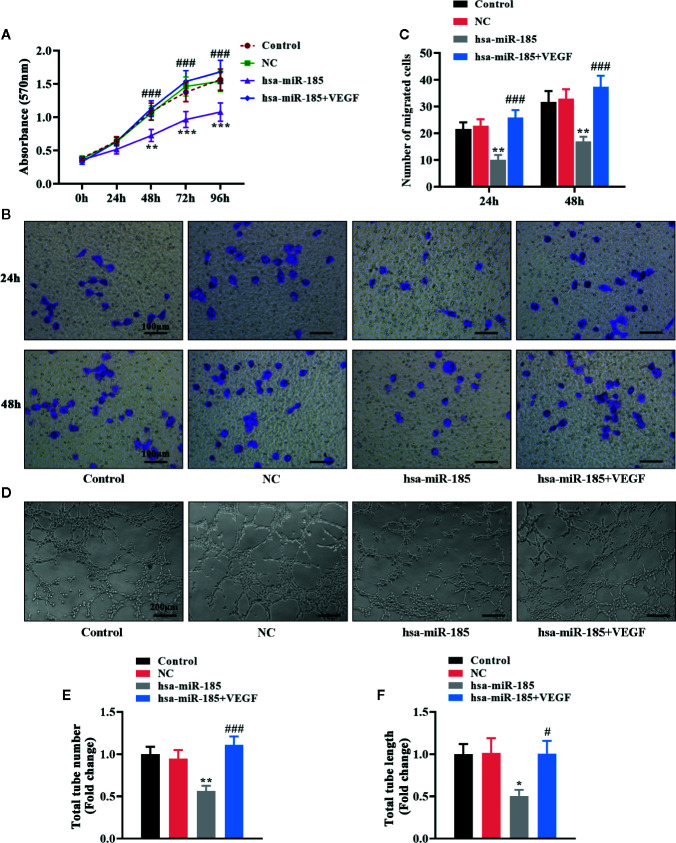
VEGF mediated the inhibition of miR-185 on cell proliferation, migration and tube formation in HOMECs. **(A)** Cell viability in HOMECs was measured using MTT assay. **(B, C)** Transwell assay was performed to evaluate the migratory ability in HOMECs. **(D–F)** Matrigel assay in HOMECs **(D)** was used to assess the relative changes about the number **(E)** and length **(F)** of total tubes. *p < 0.05, **p < 0.01, ***p < 0.001 vs NC. ^#^p < 0.05, ^###^p < 0.001 vs hsa-miR-185.

Considering the target relationships between miR-185 and VEGFA, we further investigated the role of VEGF in the angiogenic effect of miR-185 on HOMECs. The statistical curves in **Figure 6A** revealed that miR-185 could reduce the viable number of HOMECs, and VEGF treatment reversed it. Transwell assay showed that VEGF reversed the decrease of migratory cells induced by miR-185 overexpression (**Figures 6B, C**). Furthermore, we noticed that the reduction of formatted tubes in miR-185-treated HOMECs were attenuated by VEGF administration (**Figure 6D**). Accordingly, miR-185-induced decrease of total tube number was increased by VEGF (**Figure 6E**). Similar alterations were also observed in the assessment for the length of total tubes (**Figure 6F**). Totally, these results suggested that VEGF participated in the regulation of angiogenesis in vascular endothelial cells mediated by miR-185.

## Discussion

PCOS is a heterogeneous disease with ovarian dysfunction, and its etiology is still not well understood. Herein, we found that miR-185 level was decreased in the ovary of PCOS rat model and its overexpression decreased insulin levels, improved ovarian histological lesions, and blocked angiogenic processes in PCOS rats. Following the luciferase assay, VEGFA was confirmed to be a direct target gene of miR-185. Our results showed that the improvement of miR-185 on PCOS progression might be attributed to the inhibition of excessive angiogenesis by targeting VEGFA.

In general, PCOS symptoms were mainly characterized by hyperinsulinemia, insulin resistance as well as ovarian injury (Dunaif et al., 1989; Diamanti-Kandarakis and Papavassiliou, 2006). In this study, high serum insulin concentration was observed in rats after DHEA injection, indicating the occurrence of hyperinsulinemia. We also found that DHEA treatment increased the insulin resistance index and exacerbated ovarian morphological injury. Together these results suggested a well-established PCOS rat model using DHEA in our experiments.

Growing evidence showed an abnormal expression profile of miRNAs in granulose cells, theca cells, follicular fluid, adipose tissue and blood of PCOS women (Sorensen et al., 2014; Chen B. et al., 2019). For instance, miR-28-5p was revealed to potentially attenuate the pathogenesis of PCOS through interacting with prokineticin-1 (Meng et al., 2019). Teng et al. demonstrated that miR-409 was down-expressed in the ovary of PCOS rats, and its induction improved the clinical phenotypes by regulating hormone levels and pregnancy rates (Teng et al., 2019). However, limited literature had reported the association between miR-185 and PCOS development. In this work, we observed low expression of miR-185 in the ovary of PCOS rats. Similarly, the findings also demonstrated that miR-185 expression level was reduced in the cumulus granulose cells of PCOS patients (Xu et al., 2015). Thus we hypothesized that miR-185 potentially functioned as a protector in the regulation of PCOS progress. Previous studies demonstrated that miR-185 protected against low insulin sensitivity and liver steatosis in non-alcoholic fatty liver disease (Wang et al., 2014). It was also be found in hepatic cells by Chen et al. that miR-185 was an important player in improving insulin resistance (Chen D. L. et al., 2019). In consistent with these findings, we also noticed that these alterations in PCOS rats were mediated by miR-185. The results showed that miR-185 might present a therapeutic effect on PCOS progress.

It was worth noting that ovarian angiogenesis was a critical characteristic in PCOS pathogenesis (Agrawal et al., 1998; Artini et al., 2006). The angiogenic-associated factors had been proved to firmly correct the vasculature formation and regression (Ribatti et al., 2011). In angiogenic processes, two families of angiogenic factors, Angiopoietin (ANGPT) and Platelet-Derived Growth Factor (PDGF), had implications in the mature, stability and permeability of blood vessels (Hellberg et al., 2010; Fagiani and Christofori, 2013). It had been elucidated that ANGPT1 mainly promoted the formation of newly vessels, whereas ANGPT2 was responsible for vascular destabilization and regression (Davis et al., 1996; Gale et al., 2002). Abramovich et al. described that the agonist ANGPT1 was increased, and the antagonist ANGPT2 were decreased in the ovary of PCOS rat model (Abramovich et al., 2012). Furthermore, decreased expressions of ovarian PDGF proteins, like PDGFB and PDGFD, in PCOS were shown to be linked with the deregulation of ovarian angiogenesis and follicular development (Di Pietro et al., 2015; Di Pietro et al., 2016). In addition, the critical role of VEGF in vascular endothelial cell proliferation and migration was reported to affect the normal and abnormal angiogenesis (Carmeliet, 2003). In accord with these previous studies, similar alterations of angiogenic factors were also detected in the ovary of PCOS rats. Overexpression of miR-185 partially restored these alterations, which indicated that miR-185 might protect against increased angiogenesis in PCOS. VEGFA gene with rs3025020 was suggested to be associated with VEGF production to regulate the pathogenesis of PCOS (Almawi et al., 2016). Considering that the increased VEGFA in PCOS rat model was suppressed by miR-185, we questioned whether VEGFA was a downstream target of miR-185 to affect angiogenic processes. Luciferase reporter assay demonstrated that VEGFA was a direct target of miR-185, and its expression was negatively correlated with miR-185. Therefore the results indicated that the role of miR-185 in altering angiogenic factors might be mediated by interacting with VEGFA.

Endothelial cells were essential for normal structural and functional changes of vascular system (Rohlenova et al., 2018). VEGFA was demonstrated to be generally derived from stromal cells and exerted physiological functions in angiogenesis and vascular permeability to stimulate endothelial cell proliferation and migration (Weis and Cheresh, 2005; Shibuya, 2013). Our *in vitro* results further showed that VEGF overexpression could reverse the inhibitory effect of miR-185 on cell proliferation, migration and tube formation in vascular endothelial cells. Together these findings suggested that miR-185 protected against PCOS progress through inhibiting angiogenesis *via* targeting VEGFA.

In conclusion, this study shows that miR-185 is down-expressed in the ovary of PCOS rats, and its overexpression can attenuate insulin resistance, high serum insulin level and ovarian histological lesion in PCOS. Furthermore, the *in vitro* experiments demonstrate that miR-185 controls angiogenic-associated factor expression, endothelial cell proliferation and migration, as well as tube formation by directly interacting with VEGFA. Taken together, this is the first study to suggest that miR-185 acts as a protective effector on PCOS development through the inhibition of excessive angiogenesis by targeting VEGFA, which provides critical insights into understanding the etiology and pathology in PCOS.

## Data Availability Statement

The raw data supporting the conclusions of this article will be made available by the authors, without undue reservation, to any qualified researcher.

## Ethics Statement

The animal study was reviewed and approved by Shengjing Hospital of China Medical University.

## Author Contributions

YZ conceived the research, and contributed new reagents. JW conducted the experiments and analyzed the data. JW wrote the manuscript, and YZ revised the manuscript. All authors contributed to the article and approved the submitted version.

## Conflict of Interest

The authors declare that the research was conducted in the absence of any commercial or financial relationships that could be construed as a potential conflict of interest.

## Supplementary Material

Supplementary Figure 1 Representative images of green fluorescence in the ovaries of PCOS rats infected with NC or rno-miR-185 overexpressing lentivirus.

## References

[B1] Abramovich D. Irusta G. Bas D. Cataldi N. I. Parborell F. Tesone M. (2012). Angiopoietins/TIE2 system and VEGF are involved in ovarian function in a DHEA rat model of polycystic ovary syndrome. Endocrinology 153 (7), 3446–3456. 10.1210/en.2012-1105 22577112

[B2] Agrawal R. Conway G. Sladkevicius P. Tan S. L. Engmann L. Payne N. (1998). Serum vascular endothelial growth factor and Doppler blood flow velocities in in vitro fertilization: relevance to ovarian hyperstimulation syndrome and polycystic ovaries. Fertil. Steril. 70 (4), 651–658. 10.1016/s0015-0282(98)00249-0 9797093

[B3] Almawi W. Y. Gammoh E. Malalla Z. H. Al-Madhi S. A. (2016). Analysis of VEGFA Variants and Changes in VEGF Levels Underscores the Contribution of VEGF to Polycystic Ovary Syndrome. PloS One 11 (11), e0165636. 10.1371/journal.pone.0165636 27846231 PMC5112863

[B4] Ambros V. (2001). microRNAs: tiny regulators with great potential. Cell 107 (7), 823–826. 10.1016/s0092-8674(01)00616-x 11779458

[B5] Artini P. G. Monti M. Matteucci C. Valentino V. Cristello F. Genazzani A. R. (2006). Vascular endothelial growth factor and basic fibroblast growth factor in polycystic ovary syndrome during controlled ovarian hyperstimulation. Gynecol. Endocrinol. 22 (8), 465–470. 10.1080/09513590600906607 17012110

[B6] Carmeliet P. (2003). Angiogenesis in health and disease. Nat. Med. 9 (6), 653–660. 10.1038/nm0603-653 12778163

[B7] Chen B. Xu P. Wang J. Zhang C. (2019). The role of MiRNA in polycystic ovary syndrome (PCOS). Gene 706, 91–96. 10.1016/j.gene.2019.04.082 31054362

[B8] Chen D. L. Shen D. Y. Han C. K. Tian Y. (2019). LncRNA MEG3 aggravates palmitate-induced insulin resistance by regulating miR-185-5p/Egr2 axis in hepatic cells. Eur. Rev. Med. Pharmacol. Sci. 23 (12), 5456–5467. 10.26355/eurrev_201906_18215 31298399

[B9] Davis S. Aldrich T. H. Jones P. F. Acheson A. Compton D. L. Jain V. (1996). Isolation of angiopoietin-1, a ligand for the TIE2 receptor, by secretion-trap expression cloning. Cell 87 (7), 1161–1169. 10.1016/s0092-8674(00)81812-7 8980223

[B10] Di Pietro M. Parborell F. Irusta G. Pascuali N. Bas D. Bianchi M. S. (2015). Metformin regulates ovarian angiogenesis and follicular development in a female polycystic ovary syndrome rat model. Endocrinology 156 (4), 1453–1463. 10.1210/en.2014-1765 25590243

[B11] Di Pietro M. Scotti L. Irusta G. Tesone M. Parborell F. Abramovich D. (2016). Local administration of platelet-derived growth factor B (PDGFB) improves follicular development and ovarian angiogenesis in a rat model of Polycystic Ovary Syndrome. Mol. Cell Endocrinol. 433, 47–55. 10.1016/j.mce.2016.05.022 27256152

[B12] Di Pietro M. Pascuali N. Parborell F. Abramovich D. (2018). Ovarian angiogenesis in polycystic ovary syndrome. Reproduction 155 (5), R199–R209. 10.1530/REP-17-0597 29386378

[B13] Diamanti-Kandarakis E. Papavassiliou A. G. (2006). Molecular mechanisms of insulin resistance in polycystic ovary syndrome. Trends Mol. Med. 12 (7), 324–332. 10.1016/j.molmed.2006.05.006 16769248

[B14] Dunaif A. Segal K. R. Futterweit W. Dobrjansky A. (1989). Profound peripheral insulin resistance, independent of obesity, in polycystic ovary syndrome. Diabetes 38 (9), 1165–1174. 10.2337/diab.38.9.1165 2670645

[B15] Duncan W. C. Nio-Kobayashi J. (2013). Targeting angiogenesis in the pathological ovary. Reprod. Fertil. Dev. 25 (2), 362–371. 10.1071/RD12112 22951108

[B16] Fagiani E. Christofori G. (2013). Angiopoietins in angiogenesis. Cancer Lett. 328 (1), 18–26. 10.1016/j.canlet.2012.08.018 22922303

[B17] Ferrara N. Davis-Smyth T. (1997). The biology of vascular endothelial growth factor. Endocr. Rev. 18 (1), 4–25. 10.1210/edrv.18.1.0287 9034784

[B18] Forstner A. J. Basmanav F. B. Mattheisen M. Bohmer A. C. Hollegaard M. V. Janson E. (2014). Investigation of the involvement of MIR185 and its target genes in the development of schizophrenia. J. Psychiatry Neurosci. 39 (6), 386–396. 10.1503/jpn.130189 24936775 PMC4214873

[B19] Gale N. W. Thurston G. Hackett S. F. Renard R. Wang Q. McClain J. (2002). Angiopoietin-2 is required for postnatal angiogenesis and lymphatic patterning, and only the latter role is rescued by Angiopoietin-1. Dev. Cell 3 (3), 411–423. 10.1016/S1534-5807(02)00217-4 12361603

[B20] Garcia-Velasco J. A. Pellicer A. (2003). New concepts in the understanding of the ovarian hyperstimulation syndrome. Curr. Opin. Obstet. Gynecol. 15 (3), 251–256. 10.1097/01.gco.0000072860.73466.6e 12858114

[B21] Geva E. Jaffe R. B. (2000). Role of vascular endothelial growth factor in ovarian physiology and pathology. Fertil. Steril. 74 (3), 429–438. 10.1016/s0015-0282(00)00670-1 10973633

[B22] Hellberg C. Ostman A. Heldin C. H. (2010). PDGF and vessel maturation. Recent Results Cancer Res. 180, 103–114. 10.1007/978-3-540-78281-0_7 20033380

[B23] Lei G. S. Kline H. L. Lee C. H. Wilkes D. S. Zhang C. (2016). Regulation of Collagen V Expression and Epithelial-Mesenchymal Transition by miR-185 and miR-186 during Idiopathic Pulmonary Fibrosis. Am. J. Pathol. 186 (9), 2310–2316. 10.1016/j.ajpath.2016.04.015 27392970 PMC5012465

[B24] Li B. B. Li D. L. Chen C. Liu B. H. Xia C. Y. Wu H. J. (2016). Potentials of the elevated circulating miR-185 level as a biomarker for early diagnosis of HBV-related liver fibrosis. Sci. Rep. 6, 34157. 10.1038/srep34157 27677421 PMC5039723

[B25] McClure N. Healy D. L. Rogers P. A. Sullivan J. Beaton L. Haning R. V. Jr. (1994). Vascular endothelial growth factor as capillary permeability agent in ovarian hyperstimulation syndrome. Lancet 344 (8917), 235–236. 10.1016/s0140-6736(94)93001-5 7913160

[B26] Meng L. Yang H. Jin C. Quan S. (2019). miR-28-5p suppresses cell proliferation and weakens the progression of polycystic ovary syndrome by targeting prokineticin1. Mol. Med. Rep. 20 (3), 2468–2475. 10.3892/mmr.2019.10446 31322191

[B27] Palanisamy K. Nareshkumar R. N. Sivagurunathan S. Raman R. Sulochana K. N. Chidambaram S. (2019). Anti-angiogenic effect of adiponectin in human primary microvascular and macrovascular endothelial cells. Microvasc. Res. 122, 136–145. 10.1016/j.mvr.2018.08.002 30144414

[B28] Peitsidis P. Agrawal R. (2010). Role of vascular endothelial growth factor in women with PCO and PCOS: a systematic review. Reprod. BioMed. Online 20 (4), 444–452. 10.1016/j.rbmo.2010.01.007 20156703

[B29] Reynolds L. P. Grazul-Bilska A. T. Redmer D. A. (2002). Angiogenesis in the female reproductive organs: pathological implications. Int. J. Exp. Pathol. 83 (4), 151–163. 10.1046/j.1365-2613.2002.00277.x 12485460 PMC2517679

[B30] Ribatti D. Nico B. Crivellato E. (2011). The role of pericytes in angiogenesis. Int. J. Dev. Biol. 55 (3), 261–268. 10.1387/ijdb.103167dr 21710434

[B31] Rohlenova K. Veys K. Miranda-Santos I. De Bock K. Carmeliet P. (2018). Endothelial Cell Metabolism in Health and Disease. Trends Cell Biol. 28 (3), 224–236. 10.1016/j.tcb.2017.10.010 29153487

[B32] Shibuya M. (2013). Vascular endothelial growth factor and its receptor system: physiological functions in angiogenesis and pathological roles in various diseases. J. Biochem. 153 (1), 13–19. 10.1093/jb/mvs136 23172303 PMC3528006

[B33] Sorensen A. E. Wissing M. L. Salo S. Englund A. L. Dalgaard L. T. (2014). MicroRNAs Related to Polycystic Ovary Syndrome (PCOS). Genes (Basel) 5 (3), 684–708. 10.3390/genes5030684 25158044 PMC4198925

[B34] Stepto N. K. Cassar S. Joham A. E. Hutchison S. K. Harrison C. L. Goldstein R. F. (2013). Women with polycystic ovary syndrome have intrinsic insulin resistance on euglycaemic-hyperinsulaemic clamp. Hum. Reprod. 28 (3), 777–784. 10.1093/humrep/des463 23315061

[B35] Takahashi Y. Forrest A. R. Maeno E. Hashimoto T. Daub C. O. Yasuda J. (2009). MiR-107 and MiR-185 can induce cell cycle arrest in human non small cell lung cancer cell lines. PloS One 4 (8), e6677. 10.1371/journal.pone.0006677 19688090 PMC2722734

[B36] Teng Y. L. Lin S. Y. Yang H. Y. Meng L. H. Yu R. Zeng L. C. (2019). Effect of microRNA-409 on the pathogenesis of polycystic ovary syndrome. Eur. Rev. Med. Pharmacol. Sci. 23 (5), 1874–1881. 10.26355/eurrev_201903_17222 30915729

[B37] Wang X. C. Zhan X. R. Li X. Y. Yu J. J. Liu X. M. (2014). MicroRNA-185 regulates expression of lipid metabolism genes and improves insulin sensitivity in mice with non-alcoholic fatty liver disease. World J. Gastroenterol. 20 (47), 17914–17923. 10.3748/wjg.v20.i47.17914 25548489 PMC4273141

[B38] Wang Z. Zhai D. Zhang D. Bai L. Yao R. Yu J. (2017). Quercetin Decreases Insulin Resistance in a Polycystic Ovary Syndrome Rat Model by Improving Inflammatory Microenvironment. Reprod. Sci. 24 (5), 682–690. 10.1177/1933719116667218 27634381

[B39] Weis S. M. Cheresh D. A. (2005). Pathophysiological consequences of VEGF-induced vascular permeability. Nature 437 (7058), 497–504. 10.1038/nature03987 16177780

[B40] Xie Q. Cheng Z. Chen X. Lobe C. G. Liu J. (2017). The role of Notch signalling in ovarian angiogenesis. J. Ovarian Res. 10 (1), 13. 10.1186/s13048-017-0308-5 28284219 PMC5346233

[B41] Xu B. Zhang Y. W. Tong X. H. Liu Y. S. (2015). Characterization of microRNA profile in human cumulus granulosa cells: Identification of microRNAs that regulate Notch signaling and are associated with PCOS. Mol. Cell Endocrinol. 404, 26–36. 10.1016/j.mce.2015.01.030 25622783

[B42] Yu M. Liang W. Xie Y. Long Q. Cheng X. Liao Y. H. (2016). Circulating miR-185 might be a novel biomarker for clinical outcome in patients with dilated cardiomyopathy. Sci. Rep. 6, 33580. 10.1038/srep33580 27645404 PMC5028782

