# Supplementary figures and images for: MiR-185-5p Protects Against Angiogenesis in Polycystic Ovary Syndrome by Targeting VEGFA
Source: Front Pharmacol. 2020 Jul 15;11:1030. doi: 10.3389/fphar.2020.01030 (PMC7373746; doi:10.3389/fphar.2020.01030)

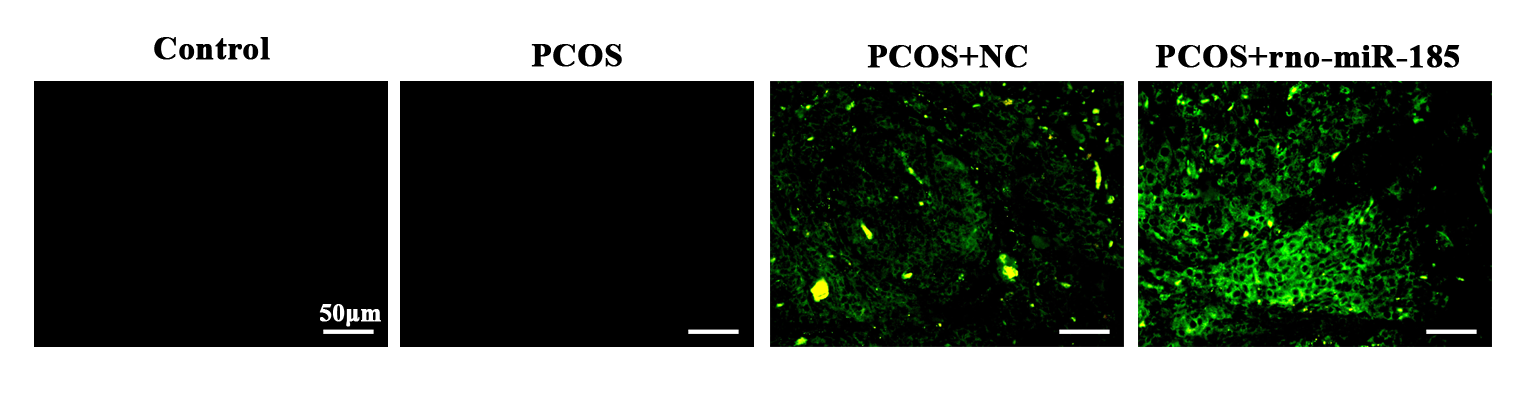

Supplement: Supplementary Figure 1 — Representative images of green fluorescence in the ovaries of PCOS rats infected with NC or rno-miR-185 overexpressing lentivirus. [file Image_1.tif]
